# Supplementary material for: Rapid fast-delta decay following prolonged wakefulness marks a phase of wake-inertia in NREM sleep
Source: Nat Commun. 2020 Jun 19;11:3130. doi: 10.1038/s41467-020-16915-0 (PMC7305232; doi:10.1038/s41467-020-16915-0)
Supplement: Supplementary file 1 — Supplementary Information [file 41467_2020_16915_MOESM1_ESM.pdf]

Supplementary information to:

Rapid fast-delta decay following prolonged wakefulness marks a phase of wake-inertia in NREM sleep

Jeffrey Hubbard, Thomas C. Gent, Marieke M. B. Hoekstra, Yann Emmenegger, Valerie Mongrain, Hans-Peter Landolt, Antoine R. Adamantidis, Paul Franken

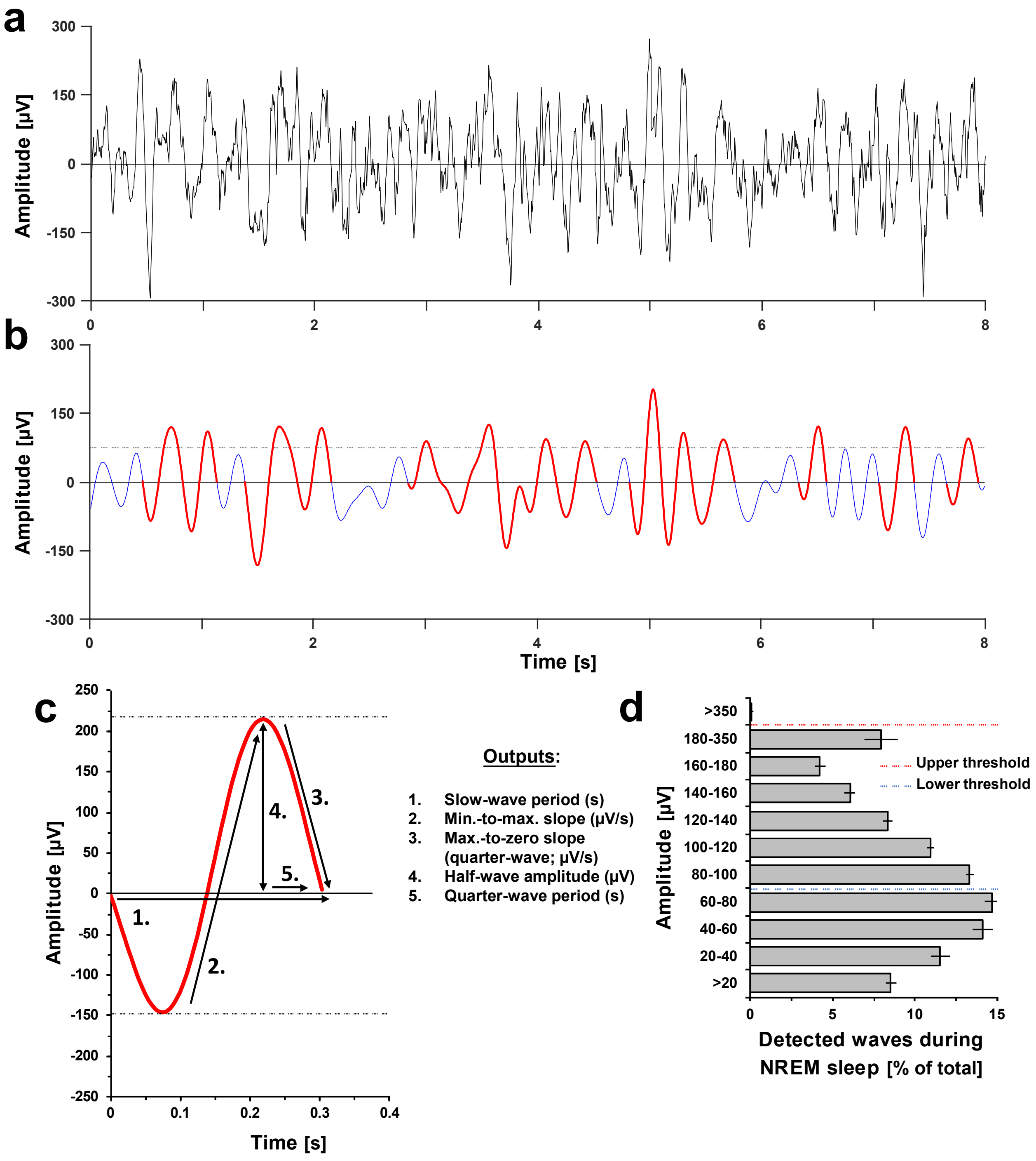

Supplementary Figure 1: Slow-wave detection algorithm

(**a**) 8-second raw EEG trace during NREMS. (**b**) EEG signal was filtered using a Chebyshev type II filter, and a zero-crossing algorithm was employed to detect slow waves (SWs). Waves of higher amplitudes than the threshold [individual mean maximum amplitude (95th percentile) of waveforms during REM sleep; dashed line; lower threshold in d] were considered SWs (highlighted in red). A maximum threshold was set at 6 times the standard deviation of the mean

positive amplitude of the filtered signal to remove signal artifacts. (**c**) Selected SWs were then analyzed for amplitude, period, and slope. (**d**) Distribution of waveforms by amplitude (at 20μV-bin resolution; except the highest 180-350μV bin) during NREMS expressed as a total of all detected waves across 48h of baseline and 42h of recovery, with maximum (red dashed line) and minimum (blue dashed line) threshold values (n=37). Values in d are expressed as mean ± s.e.m. Further details are available in the Methods section.

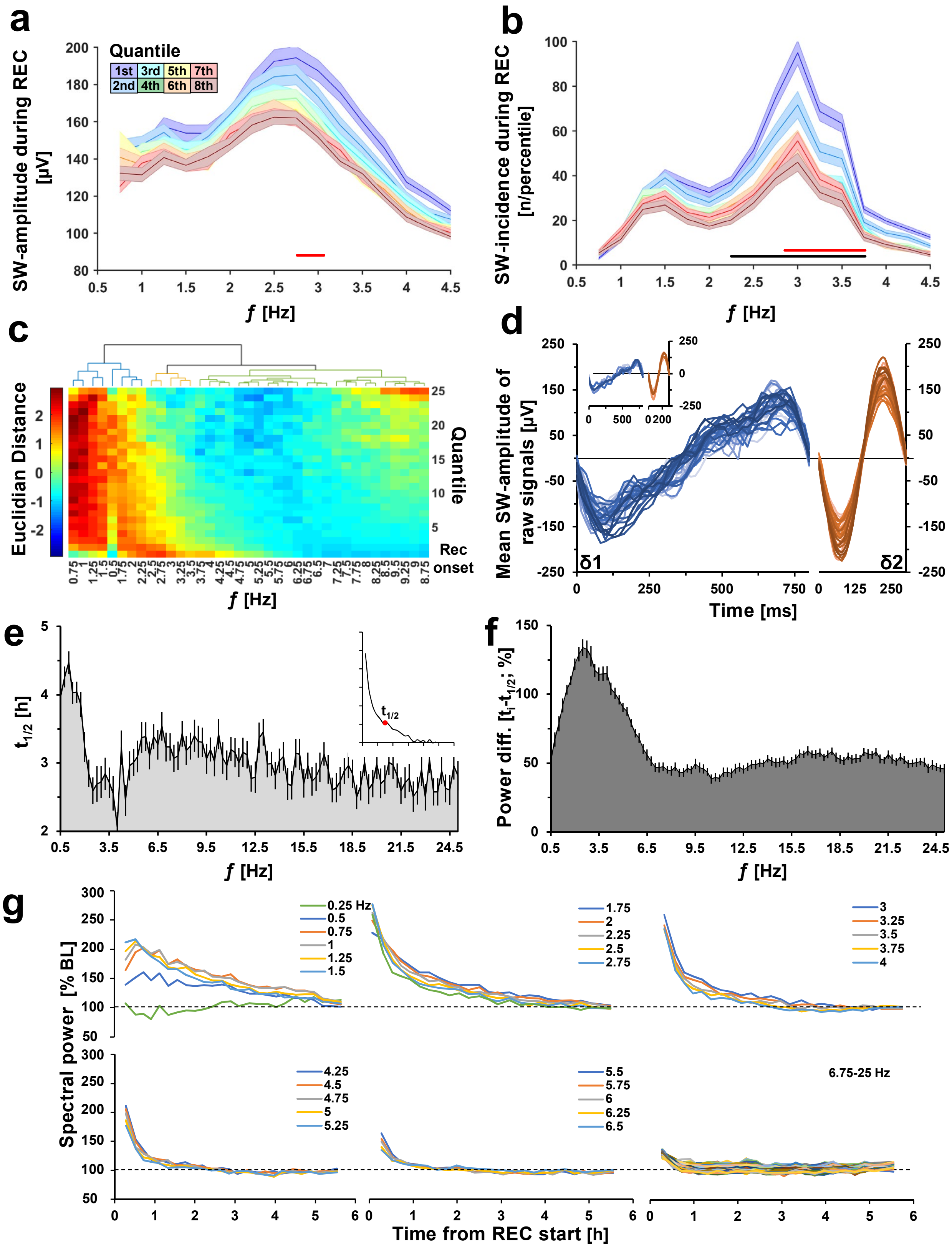

Supplementary Figure 2: Defining the δ1- and δ2-frequency bands

(**a**) Mean SW-amplitude of filtered signals per 0.25 Hz bin of detected SWs during the first 8 out of 25 NREMS time quantiles over the 1st 6h of recovery (REC) after sleep deprivation (SD; as in Fig. 2) (2-way rANOVA factors quantile x frequency; 1st-2nd quantile: $F_{15,930}$=3.9, p=8.9E-12; red line denote post-hoc t-test p<0.05). (**b**) Uncorrected mean SW-incidence binned per 0.25 Hz (as in Fig. 2B) (2-way rANOVA factors quantile x frequency; 1st-2nd: $F_{15,1080}$=5.8, p=8.9E-12; 2nd-3rd: $F_{15,930}$=3.3, p=2.0E-5). Post-hoc significance (p<0.05) between 1st-2nd and 2nd-3rd quantiles are indicated respectively in red and black bars below. For A and B n=37. (**c**) Unsupervised hierarchical clustering of EEG spectral (0.5-9.5 Hz) dynamics over the 1st 6 h of recovery grouped 0.25 Hz dynamics into δ1- (0.5-1.75 Hz; blue) and δ2- (2.5-3.5 Hz; orange) bands, and higher frequencies (3.75-9.5 Hz; green; see n=38) according to the dendrogram on top. Darker red indicates closer association, expressed as Euclidian distance to the mean. Note right-hand time axis denotes NREMS quantiles (1-25) increasing from bottom to top. (**d**) As in Figure 2F, average waveforms of unfiltered (raw) detected δ1- (blue-left) and δ2- (orange-right) waves during the 1st 10 minutes of recovery NREMS, for individual mice using a frontal-parietal derivation (n=37). Note that the fronto-cerebellar derivation yields similar average waveforms (insert; n=6). (**e**) Time required for power density in the respective frequency bins to reach half of its initial value during recovery ($t_{1/2}$). Higher frequencies (>4.5 Hz) reach $t_{1/2}$ faster than the slowest (<2 Hz), however initial values start much lower and thus decay less overall (ratio difference; Fig. S3F). (**g**) Average dynamics of EEG spectra at 0.25Hz resolution across the 1st 6 h of recovery expressed in 25 NREMS time quantiles (n=38). Higher initial values are observed as frequency increases within the δ-band. Note in <1.75 Hz power density initially increases before decreasing. Bars in e,f and shading in a,b represents ± s.e.m.

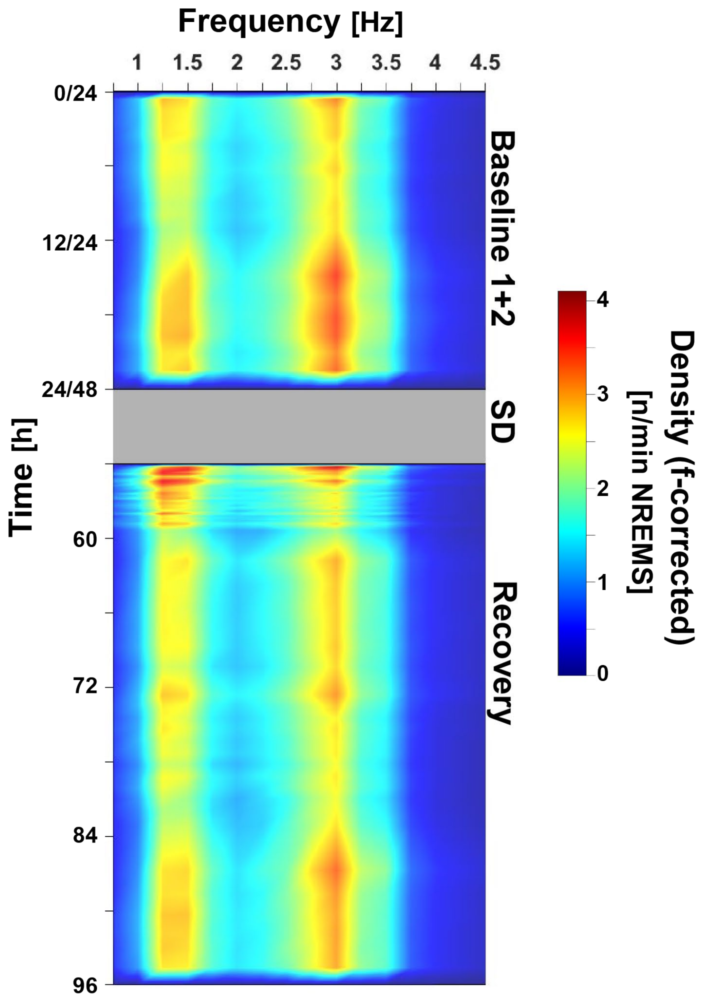

Supplementary Figure 3: Bimodal distribution of SWs across baseline and recovery
Heat-plot of mean frequency-corrected SW-density distribution (0.5-4.5 Hz) during baseline (2-day average) and during recovery from sleep deprivation (SD; grey rectangle). Note the ubiquitous presence of the bimodal distribution centered at 1.25 and 3.0 Hz, respectively. Data are presented using the same NREMS time quantiles as in Figure 1 (n=37).

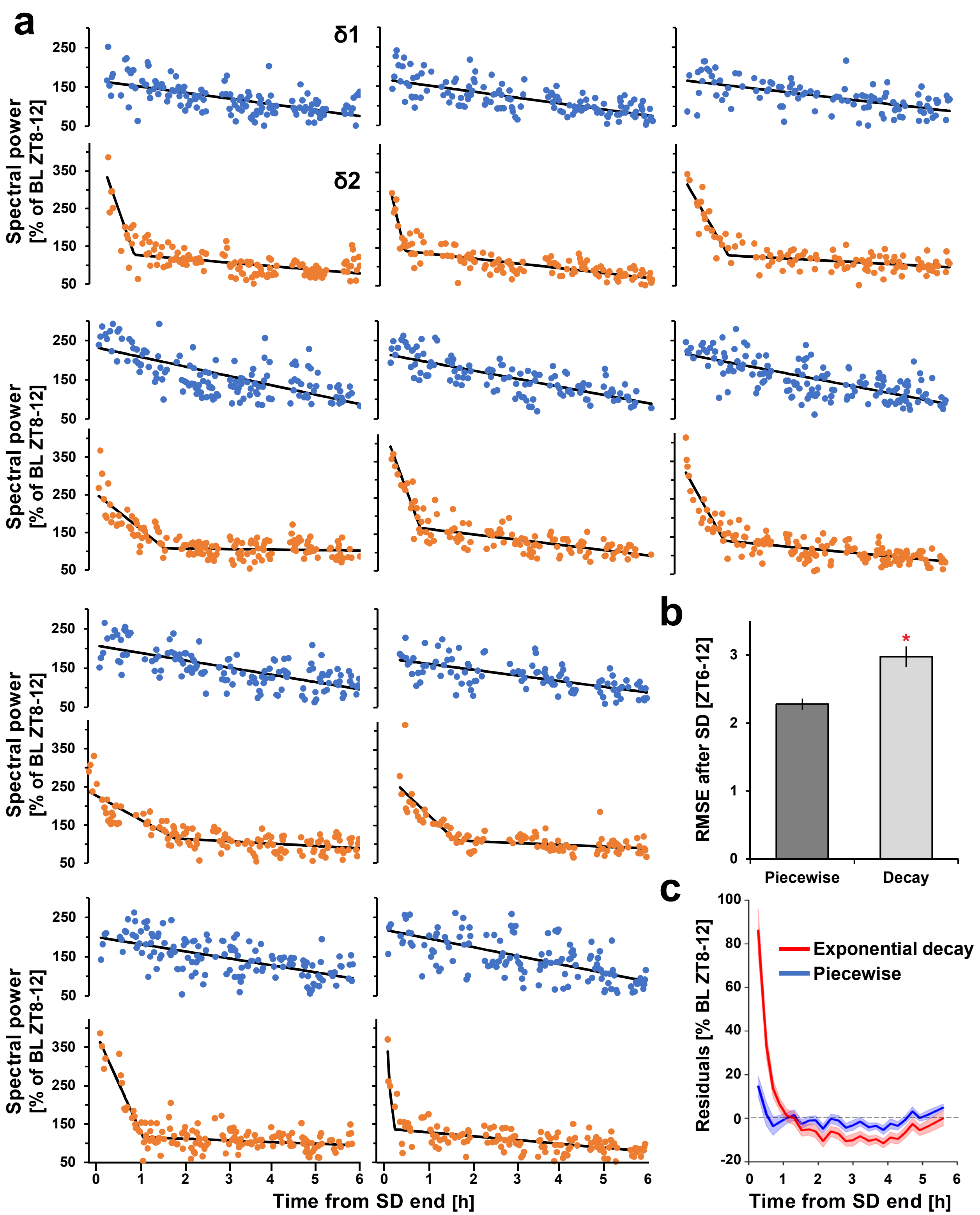

Supplementary Figure 4: Comparison of δ1- and δ2-decreases during recovery NREMS

(**a**) Examples of mean δ1- (blue) and δ2- (orange) power per NREMS episode (>32s) during the 1st 6h of recovery for 10 individual mice. Note the rapid decrease in δ2 for most mice during initial episodes, and the overall linear trend of δ1. (**b**) Comparison of root mean square of errors (RMSE)

values for 2-segment piecewise regression vs. exponential decay for δ2-changes across the 1st 6h following sleep deprivation using individual NREMS episodes. RMSE was lower for the piecewise regression model, indicating a better fit (paired t-test: t_{37}=-6.0, p=6.1E-07, red asterisk denotes significance). (**c**) Residuals for the same regression models. Note that in order to fit the steep initial decline and subsequent abrupt change to a shallow linear decay, the exponential decay had to greatly overestimate the initial values and underestimate those following pivot-point. Bars in B and shading in C represent s.e.m. (n=38). Coefficient estimates for exponential decay model: $\delta 2_{t0}$=341.2±27.3%, time-constant=0.96±0.15h, lower asymptote=99.9±2.5%. See Results for estimates of the piece-wise regression.

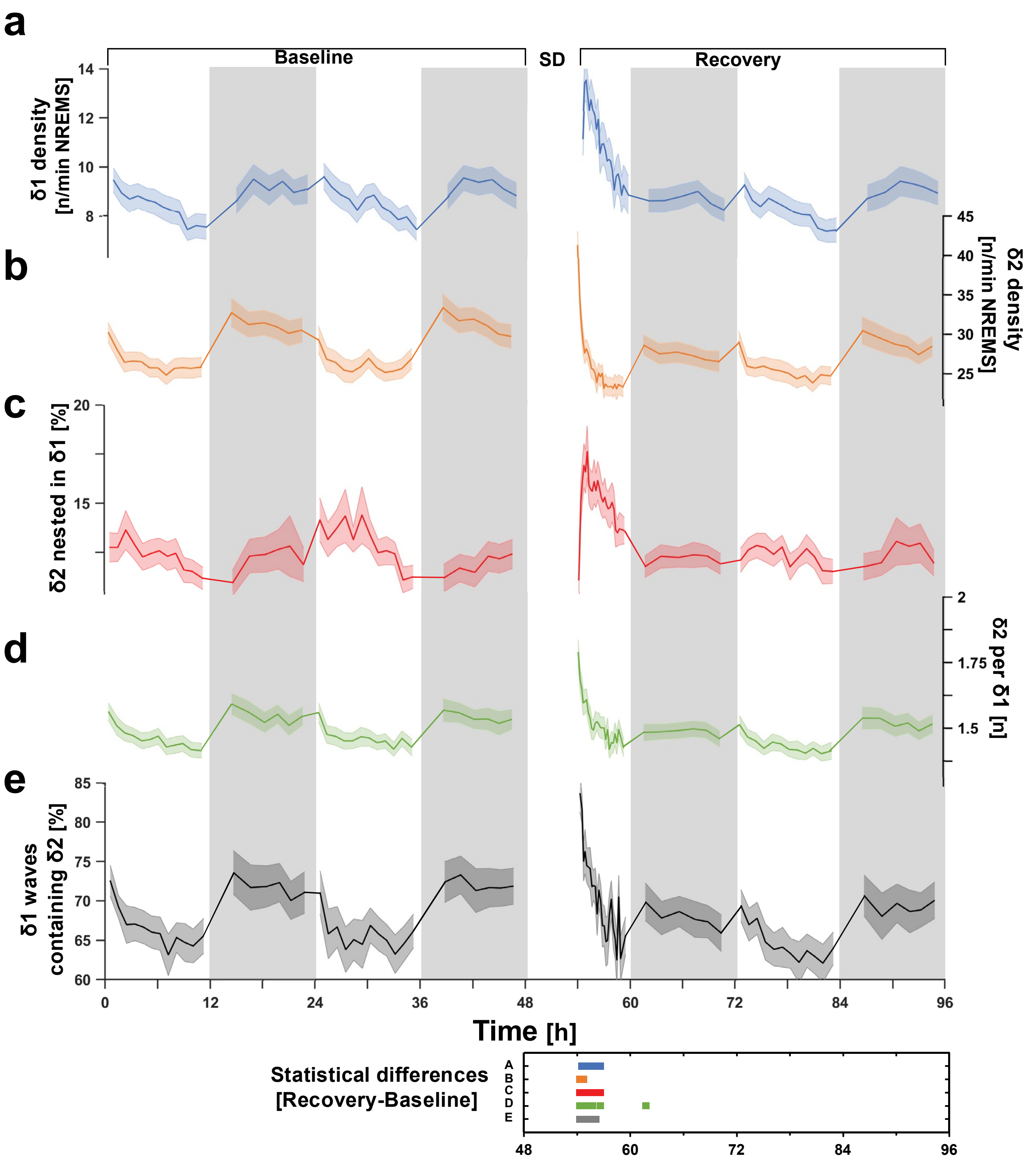

Supplementary Figure 5: Nested δ2-waves are present at all times during baseline and recovery

Density of detected NREMS SWs within the δ1- (**a**) and δ2- (**b**) range across baseline and recovery from sleep deprivation (SD) (2-way rANOVA factors SD x time; δ1: $F_{34,1836}$=26.3, p<1.0E-17; δ2: $F_{34,1836}$=19.2, p<1.0E-17). Note the similarity with the time dynamics of spectral power in the two bands following SD (Figs. 4a, 7a). (**c**) Percentage of total detected δ2-waves from filtered EEG (2-

4.5 Hz), which are co-localized (nested) within a δ1-wave (2-way rANOVA factors SD x time: $F_{34,1836}$=7.5, p<1.0E-17). (**d**) Average number of δ2-waves nested in δ1. Note the increase following spontaneous waking (baseline dark) and SD (2-way rANOVA factors SD x time: $F_{34,1836}$=11.5, p<1.0E-17). (**e**) This is mirrored in the percentage of δ1-waves which contain nested δ2-waves (2-way rANOVA factors SD x time: $F_{34,1836}$=9.5, p<1.0E-17). Data are presented using the same quantiles as in Figure 1. Values represent mean ± s.e.m. Significant post-hoc differences (p<0.05) between averaged baseline days and each recovery day following rANOVA are presented in a color-matched panel below and letters (a-e) refer to the variables depicted in their respective panels (n=37). Overall dynamics in a,b are comparable to Supp. Fig. 6b.

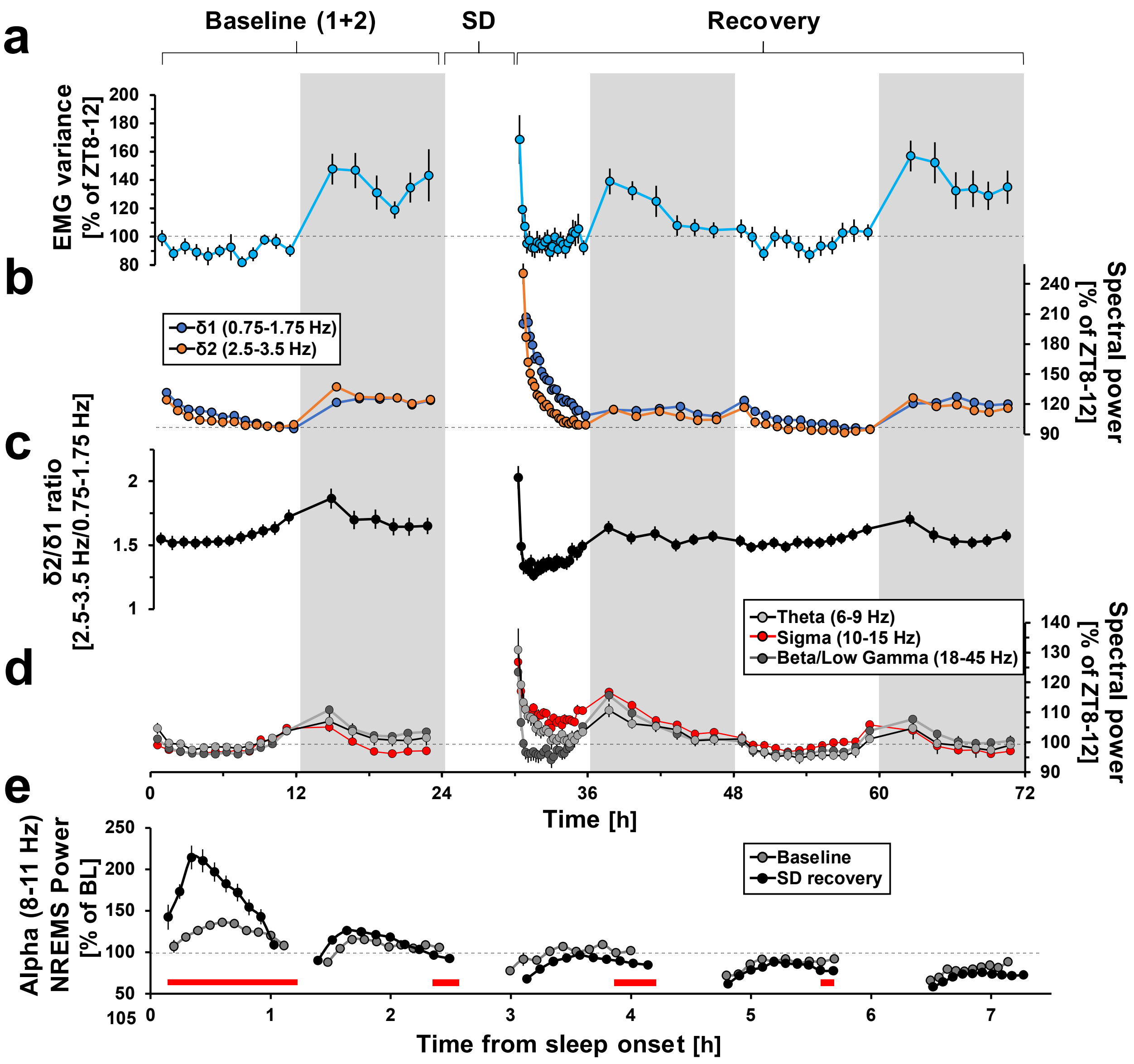

Supplementary Figure 6: Initial changes across different in EEG/EMG measures activity consecutive to prolonged waking reflect a NREMS sub-state

Higher levels in EMG variance (**a**), δ1- and δ2-power (**b**), δ2/δ1-power ratio (**c**), and other spectral EEG components [Theta (grey), Sigma (red), and Beta/Low Gamma (dark-grey)] (**d**), are reached not only after sleep deprivation (SD), but also after prolonged spontaneous waking in the dark periods of baseline and 2nd recovery day. As in Figs. 4a and 7a. Initial recovery values differ significantly from baseline for these three measures. For a-d n=38. (**e**) NREMS EEG alpha activity (8-11 Hz) during baseline (grey circles) and recovery (black circles) in humans (2-way rANOVA factors condition x time-course: $F_{49,10535}$=8.93, p<1.0E-17; n=110). Red lines indicate significant post-hoc differences between baseline and recovery (post-hoc paired t-tests, p<0.05). Data in all panels is presented as mean values ± s.e.m.

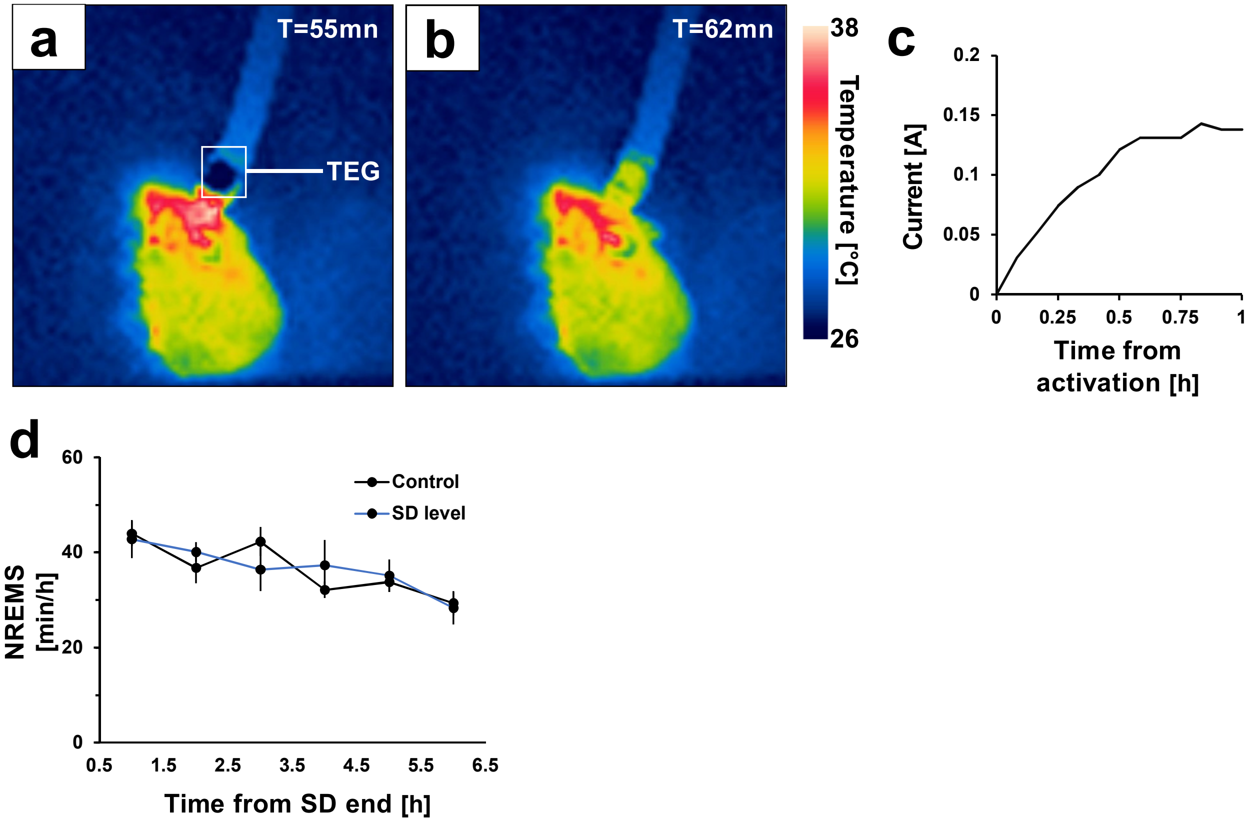

Supplementary Figure 7: Confirmation of TEG efficacy during cortical warming

Thermal image of one mouse undergoing cortical warming during (**a**) and 2 minutes after the thermo-electric generator (TEG) has been deactivated (**b**). The cooled side of the TEG is visible as a dark-blue boxed-in area above the EEG connector in a, which begins to reach the animal's surface temperatures after deactivation in **b**. (**c**) Example in one mouse of the increase in current (amps; **a**) needed to compensate for decreases in cortical temperature during the manipulation. (**d**) NREMS amounts for control (recovery without warming) and recovery with warming in which cortical temperature was kept at sleep-deprivation levels (SD level) for the first recovery hour. Data is presented as means ± s.e.m (n=4).

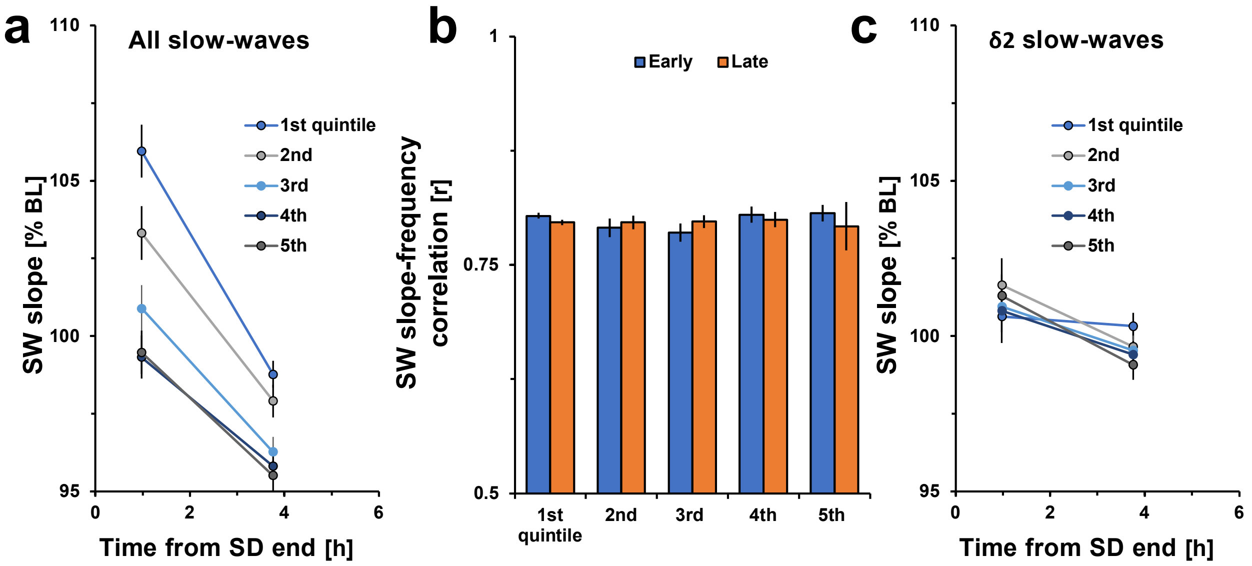

Supplementary Figure 8: Changes in SW-slope with amplitude binning in 'early' and 'late' recovery NREMS depend on changes in δ2-waves

Sleep-wake driven changes in SW-slope (corrected for amplitude) are thought to reflect an underlying process of synaptic strengthening during waking and of synaptic downscaling during NREMS[1]. The increase in SW-slope after sleep deprivation (SD) can, however, be explained in large part by taking into account that prolonged waking predominantly increases amplitude and prevalence of δ2-waves, which have steeper slopes than δ1-waves of equal amplitude. (**a**) Combining all SWs (0.75-4.5 Hz), SW-slope following amplitude binning (quintiles; five 20%-bins equally spaced between minimum and maximum amplitudes for each animal), is higher in all bins in early (left; before δ pivot- point) vs. late (right data point; after pivot- point) recovery NREMS, consistent with published data[2,3]. Data are plotted as mean ± s.e.m. percentage changes from NREMS during baseline (BL; ZT8-12). (**b**) SW-slope to SW-frequency correlations during the 6h recovery show high correlations, regardless of amplitude bin. (**c**) When corrected for frequency, by re-filtering raw signals for the δ2-band (2-4.5 Hz, as in Fig. 2 h,i), SW-slopes in early and late recovery show much lower changes. Mean differences in SW-slopes between early and late recovery were significantly decreased when considering δ2-waves only (all SWs: 4.9±0.6%; δ2- waves: 1.5±0.2%; paired t-test: t_{72}=-5.7, p=2.0E-7), suggesting that the dynamic changes in SW- slope importantly depend on the SW-frequency and sleep-wake driven changes in prevalence and amplitude of δ-wave population.

Supplementary references

1 Tononi, G. & Cirelli, C. Sleep function and synaptic homeostasis. *Sleep Med Rev* **10**, 49-62, (2006).

2 Panagiotou, M., Vyazovskiy, V. V., Meijer, J. H. & Deboer, T. Differences in electroencephalographic non-rapid-eye movement sleep slow-wave characteristics between young and old mice. *Sci Rep* **7**, 43656, (2017).

3 Vyazovskiy, V. V., Cirelli, C. & Tononi, G. Electrophysiological correlates of sleep homeostasis in freely behaving rats. *Progress in brain research* **193**, 17-38, (2011).
