# Supplementary material for: Rapid fast-delta decay following prolonged wakefulness marks a phase of wake-inertia in NREM sleep
Source: Nat Commun. 2020 Jun 19;11:3130. doi: 10.1038/s41467-020-16915-0 (PMC7305232; doi:10.1038/s41467-020-16915-0)
Supplement: Supplementary file 3 — Reporting Summary [file 41467_2020_16915_MOESM3_ESM.pdf]

nature research

Corresponding author(s): Paul Franken

Last updated by author(s): May 5, 2020

Reporting Summary

Nature Research wishes to improve the reproducibility of the work that we publish. This form provides structure for consistency and transparency in reporting. For further information on Nature Research policies, see our Editorial Policies and the Editorial Policy Checklist.

Statistics

For all statistical analyses, confirm that the following items are present in the figure legend, table legend, main text, or Methods section.

n/a	Confirmed	
☐	☒	The exact sample size (*n*) for each experimental group/condition, given as a discrete number and unit of measurement
☐	☒	A statement on whether measurements were taken from distinct samples or whether the same sample was measured repeatedly
☐	☒	The statistical test(s) used AND whether they are one- or two-sided<br>*Only common tests should be described solely by name; describe more complex techniques in the Methods section.*
☒	☐	A description of all covariates tested
☐	☒	A description of any assumptions or corrections, such as tests of normality and adjustment for multiple comparisons
☐	☒	A full description of the statistical parameters including central tendency (e.g. means) or other basic estimates (e.g. regression coefficient) AND variation (e.g. standard deviation) or associated estimates of uncertainty (e.g. confidence intervals)
☐	☒	For null hypothesis testing, the test statistic (e.g. *F*, *t*, *r*) with confidence intervals, effect sizes, degrees of freedom and *P* value noted<br>*Give P values as exact values whenever suitable.*
☒	☐	For Bayesian analysis, information on the choice of priors and Markov chain Monte Carlo settings
☒	☐	For hierarchical and complex designs, identification of the appropriate level for tests and full reporting of outcomes
☐	☒	Estimates of effect sizes (e.g. Cohen's *d*, Pearson's *r*), indicating how they were calculated

Our web collection on statistics for biologists contains articles on many of the points above.

Software and code

Policy information about availability of computer code

Data collection	Data was collected using commercially available systems from Embla (EMBLA A10), Intan (RHD2000), FLIR (Model E30, FLIR Systems)
Data analysis	Sleep/wake states were scored using Somnologica (v3; Medcare Flaga; Thornton) or Rembrandt® Analysis Manager (version 8; Embla Systems, Broomfield, CO, USA). Data was analyzed using custom scripts in Matlab (v2019B, Mathworks Inc.), and binary files were generated with Pascal (v5; TMT Development corporation).

For manuscripts utilizing custom algorithms or software that are central to the research but not yet described in published literature, software must be made available to editors and reviewers. We strongly encourage code deposition in a community repository (e.g. GitHub). See the Nature Research guidelines for submitting code & software for further information.

Data

Policy information about availability of data

All manuscripts must include a data availability statement. This statement should provide the following information, where applicable:

- Accession codes, unique identifiers, or web links for publicly available datasets
- A list of figures that have associated raw data
- A description of any restrictions on data availability

The main dataset consisting of 38 male mice implanted for sleep/wake phenotyping and analysed during the current study are available in the FigShare repository, complete with data descriptors at:

Raw data can be extracted from this dataset and analyzed for Figures 1,2,3A,3D,7A,7B,7C, 7D; Supplementary Figures S1,S2,S3,S4.S5,S6A,S6B,S6C,S6D,S8A,S8B,S8C.

Other datasets analysed (tetrode and optogenetics mice data, and human EEG data) for the current study come from previously published manuscripts and are

available from the appropriate authors upon reasonable request.

All figures in the manuscript have associated excel spreadsheets to reproduce graphs.

Field-specific reporting

Please select the one below that is the best fit for your research. If you are not sure, read the appropriate sections before making your selection.

- [x] Life sciences
- [ ] Behavioural & social sciences
- [ ] Ecological, evolutionary & environmental sciences

For a reference copy of the document with all sections, see nature.com/documents/nr-reporting-summary-flat.pdf

Life sciences study design

All studies must disclose on these points even when the disclosure is negative.

Sample size	38 mice (main dataset), 33 mice (sleep deprivations given at different times), 3 mice (respiratory recordings), 4 mice (temperature manipulation), 6 mice (tetrode implants), 4 mice (optogenetic silencing), and 110 human subjects were used. As this study was a type of meta-analysis of previously published data, sample sizes were determined in these manuscripts directly.
Data exclusions	No data was excluded from the study.
Replication	In mice, experimental results were first validated against previous publications using the same animals and the same standardized phenotyping. Animals specific to this study were validated against other mice. All attempts at replication were successful.
Randomization	No randomization was required as all animals were wild-type mice, and all humans, healthy subjects.
Blinding	Scoring of sleep and wake states were performed by experienced annotators for both mice and human experiments. Analysis was done blind to the individual subjects or animals.

Reporting for specific materials, systems and methods

We require information from authors about some types of materials, experimental systems and methods used in many studies. Here, indicate whether each material, system or method listed is relevant to your study. If you are not sure if a list item applies to your research, read the appropriate section before selecting a response.

Materials & experimental systems

n/a	Involved in the study	
x		Antibodies
x		Eukaryotic cell lines
		Palaeontology and archaeology
	x	Animals and other organisms
	x	Human research participants
x		Clinical data
x		Dual use research of concern

Methods

n/a	Involved in the study	
x		ChIP-seq
x		Flow cytometry
x		MRI-based neuroimaging

Palaeontology and Archaeology

Specimen provenance	*Provide provenance information for specimens and describe permits that were obtained for the work (including the name of the issuing authority, the date of issue, and any identifying information).*
Specimen deposition	*Indicate where the specimens have been deposited to permit free access by other researchers.*
Dating methods	*If new dates are provided, describe how they were obtained (e.g. collection, storage, sample pretreatment and measurement), where they were obtained (i.e. lab name), the calibration program and the protocol for quality assurance OR state that no new dates are provided.*

- [ ] Tick this box to confirm that the raw and calibrated dates are available in the paper or in Supplementary Information.

Ethics oversight	*Identify the organization(s) that approved or provided guidance on the study protocol, OR state that no ethical approval or guidance was required and explain why not.*

Note that full information on the approval of the study protocol must also be provided in the manuscript.

Animals and other organisms

Policy information about studies involving animals; ARRIVE guidelines recommended for reporting animal research

Laboratory animals	C57B16 male mice aged 10-14 weeks (fronto-parietal EEG implantation), C57B16 male mice aged 6 weeks at time of virus injection and 10 weeks at time of EEG recording (tetrode and optogenetic silencing).
Wild animals	No wild animals were used in the study.
Field-collected samples	No field-collected samples were used in the study.
Ethics oversight	Animal studies were approved by the Cantonal Veterinary authorities of Vaud and Bern, in Switzerland.

Note that full information on the approval of the study protocol must also be provided in the manuscript.

Human research participants

Policy information about studies involving human research participants

Population characteristics	Healthy young male subjects, aged approximately 24 years old at the time of the study. No subject had an excluding medical diagnosis or was under pharmacological treatment.
Recruitment	Subjects were recruited through public advertisement of scientific research in the city of Zurich, Switzerland.
Ethics oversight	All human studies were approved by independent Institutional Review Boards and complied with the respective laws and regulations on research in human subjects and the WMA Declaration of Helsinki.

Note that full information on the approval of the study protocol must also be provided in the manuscript.
